# Activity of Herbal Medicines on *Plasmodium falciparum* Gametocytes: Implications for Malaria Transmission in Ghana

**DOI:** 10.1371/journal.pone.0142587

**Published:** 2015-11-12

**Authors:** Linda Eva Amoah, Courage Kakaney, Bethel Kwansa-Bentum, Kwadwo Asamoah Kusi

**Affiliations:** 1 Department of Immunology, Noguchi Memorial Institute for Medical Research, College of Health Sciences, University of Ghana, Legon, Ghana; 2 Department of Animal Biology and Conservation Science, College of Basic and Applied Sciences, University of Ghana, Legon, Ghana; Instituto Nacional de Salud Pública, MEXICO

## Abstract

**Background:**

Malaria still remains a major health issue in Ghana despite the introduction of Artemisinin-based combination therapy (ACT) coupled with other preventative measures such as the use of insecticide treated nets (ITNs). The global quest for eradication of malaria has heightened the interest of identifying drugs that target the sexual stage of the parasite, referred to as transmission-blocking drugs. This study aimed at assessing the efficacy and gametocydal effects of some commonly used herbal malaria products in Ghana.

**Methodology/Principal Findings:**

After identifying herbal anti-malarial products frequently purchased on the Ghanaian market, ten of them were selected and lyophilized. *In vitro* drug sensitivity testing of different concentrations of the herbal products was carried out on asexual and *in vitro* generated gametocytes of the 3D7 strain of *Plasmodium falciparum*. The efficacies of the products were assessed by microscopy. Cultures containing low dose of RT also produced the least number of late stage gametocytes. Two of the herbal products CM and RT inhibited the growth of late stage gametocytes by > 80% at 100 μg/ml whilst KG was the most inhibitory to early stage gametocytes at that same concentration. However at 1 μg/ml, only YF significantly inhibited the survival of late stage gametocytes although at that same concentration YF barely inhibited the survival of early stage gametocytes.

**Conclusions/Significance:**

Herbal product RT (*Aloe schweinfurthii*, *Khaya senegalensis*, *Piliostigma thonningii* and *Cassia siamea*) demonstrated properties of a highly efficacious gametocydal product. Low dose of herbal product RT exhibited the highest gametocydal activity and at 100 μg/ml, RT exhibited >80% inhibition of late stage gametocytes. However inhibition of asexual stage parasite by RT was not optimal. Improving the asexual inhibition of RT could convert RT into an ideal antimalarial herbal product. We also found that generally *C*. *sanguinolenta* containing *herbal products* exhibited gametocydal activity in addition to high asexual efficacy. Herbal products with high gametocydal activity can help in the fight to reduce malaria transmission.

## Introduction

Malaria remains a major cause of morbidity and mortality worldwide with more than 3.3 billion people worldwide at risk since they live in countries with ongoing transmission [1]. In 2013, it was estimated that there were 198 million cases of malaria worldwide, with 584,000 deaths, of which 90% occurred in Africa [1]. The sexual stages of the malaria parasite are responsible for malaria transmission. In the human host, these sexual stages begin as sexually-committed ring stage parasites, develop into early stage (I to II) gametocytes, and finally mature into late stage (III through V) gametocytes [2]. Whereas only mature stage V gametocytes are found in circulation, the immature stages (I to IV) are sequestered in the vasculature and bone marrow [3,4]. Stage V gametocytes are released from sequestration into circulation, however they only become infectious to the mosquito after they undergo a few days of additional maturation in circulation. During a blood meal, the female *Anopheles* mosquito picks up mature (stage V) gametocytes. Within the mosquito, gametocytes complete the sporogonic life cycle, which results in the mosquito becoming infectious to humans as they then harbour sporozoites within their salivary glands. These sporozoites are then passed to a new human host during a subsequent blood meal of that infectious mosquito.

The pathophysiology of malaria is mostly due to the asexual stages of the *Plasmodium* parasite within the human host [5], therefore most anti-malarial drugs target the asexual stages of the parasite, with very few targeting the transmissible stages [6]. Primaquine is currently the only licensed anti-malarial drug that is effective against late stage *Plasmodium falciparum* gametocytes but has certain drawbacks such as its tendency to cause acute haemolysis in individuals with glucose-6-phosphate dehydrogenase (G6PD) deficiency [7]. Since G6PD mutations are prevalent in West Africa, the use of primaquine is limited [8].These aside, *P*. *falciparum* parasites are continuously evolving and have gained resistance to all orthodox anti-malarial drugs, including artemisinins [9–11]. This, coupled with the fact that there is no clinical vaccine for malaria, makes the discovery and production of new anti-malarial drugs that target both the asexual and sexual stages of the parasites imperative.

The relatively high cost of orthodox medication coupled with the high cost of western medical care as well as socio-cultural practices of Ghanaians have resulted in a high percentage of the population to rely heavily on local herbal products to treat numerous aliments, including malaria [12,13]. Herbal medicines include herbs, herbal materials, herbal preparations and finished herbal products that contain parts of plants or other materials, or combinations thereof as active ingredients [14]. One major problem with herbal medicine in Ghana is the lack of data on their efficacy and toxicity. However, the recent revolution in the plant medicine industry has led to a large number of herbal products going through rebranding and packaging as well as rigorous national testing and licensing, resulting in herbal products with enhanced efficacy and reduced toxicity. Despite these rigorous testing regimens, information regarding adverse effects, counter indications as well as information on prescribed dosage for a complete course are still lacking in majority of these products. Even though a lot of research has been conducted on some individual plant active ingredients such as *Azadirachta indica* which has proven to be potent at reducing asexual parasitaemia and gametocytaemia of *P*. *falciparum* [15], not much research has been conducted on the combined effect of commonly used plant active ingredients, which could produce synergistic or antagonistic effects.

With the new worldwide agenda on the total eradication of malaria and the high consumption of herbal antimalarial products in Ghana, we decided to study the effects some of the most patronized herbal antimalarials we have on malaria transmission and possibly identify those that can reduced the transmission of malaria parasites. The main attribute for resistance to most orthodox anti-malarial drugs has been improper administration of the drug, usually as a monotherapy, and a recent report suggests that malaria parasites have a much lower tendency of gaining resistance to extracts from whole plant *Artemesia annua* as opposed to the purified product artemisinin [16], suggesting a higher tendency to gain resistance to a purified product than unpurified extract. Also knowledge from studies conducted on orthodox drugs suggests that *P*. *falciparum* parasites continuously exposed to sub optimal drug concentration results in a heightened production and spread of drug resistant parasites. The main aim of this study was therefore to screen a number of approved and commonly used local herbal anti-malarial products for potential gametocydal activities.

## Materials and Methods

### Consumer survey for anti-malarial herbal products

A consumer survey was carried out through an interview questionnaire administrated to attendants of herbal shops in Accra, the capital city of Ghana. The top ten most patronised herbal products for treating malaria, which also have been approved by the Foods and Drugs Authority (FDA) of Ghana, were chosen for the study. Details of the 10 selected herbal products are presented in Table 1.

**Table 1:** Properties of herbal anti-malarial plant products.

Product^a^ (Package size)	Dosage	Average number sold by each herbal shop/ week	Active plant ingredients	Diseases treated other than malaria
YF (330 ml)	120	31	*Cryptolepis sanguinolenta*, *Azadirachta indica*	Typhoid
CM (500 ml)	90	6	*Carapa procera*, *Cryptolepis sanguinolenta*	Typhoid
MS (330 ml)	90	62	*Cryptolepis sanguinolenta*	General weakness, stomach ulcer
AN (500 ml)	90	67	*Cola gigantean*, *Solanum torvum*, *Spathodea campanulata*, *Bombax buonopozense*, *Vernonia amygdalina*	Typhoid, jaundice, menstrual pain, general body pain, loss of appetite, candidiasis, waist pain
TB (500 ml)	45	58	*Ocimum viride*, *Azadirachta indica*, *Paullinia pinnata*, *Tetrapleura tetraptera*, *Theobroma cacao*, *Cymbopogon citratus*, *Moringa oleifera*	Typhoid, jaundice, menstrual pain, stomach pain, candidiasis, body pain, loss of appetite, stomach ulcer
AD (500 ml)	90	41	*Anthocleista nobilis*, *Vitex grandifolia*, *Phyllanthus fraternus*	Typhoid, jaundice, menstrual pain, headache
RT (500 ml)	180	73	*Aloe schweinfurthii*, *Khaya senegalensis*, *Piliostigma thonningii*, *Cassia siamea*	Typhoid, jaundice, stomach ulcer
HB (300 ml)	150	31	*Cryptolepis sanguinolenta*, *Alstonia boonei*, *Azadirachta indica*, *Monodora myristica*, *Xylopia aethiopica*	-
TF (330 ml)	90	35	*Azadirachta indica*, *Alstonia boonei*	-
KG (300 ml)	135	31	*Nauclea latifolia*, *Phyllanthus fraternus*, *Cryptolepis sanguinolenta*	-

^a^Product names are unique identifiers and not the actual names of the licensed products.

### Processing of herbal products

Ten millilitres of each herbal product was transferred into a 50 ml falcon tube under sterile conditions and kept at-80°C for 48 hours. They were then lyophilized using the LABCONCO Freezone6. Ten milligram each of freeze-dried herbal product was dissolved in 10 ml of distilled water, giving a stock concentration of 1,000 μg/ml. The stock herbal products were filter-sterilized through a 0.2 μm Acrodisc^®^ filter membrane and stored in a -20°C freezer until use.

### Culturing of *Plasmodium* parasites

The efficacy of herbal products on both sexual and asexual parasite stages was tested on the 3D7 chloroquine-sensitive strain of *P*. *falciparum*. Continuous *P*. *falciparum* asexual cultures were maintained *in vitro* using a modified method of Trager and Jensen [17]. Complete parasite media (CPM) consisted of RPMI 1640 supplemented with HEPES, L-glutamine, NaHCO_3_, glucose, gentamycin and Albumax II. Unless otherwise stated, conditions of incubation used in all the experiments were as follows; 37°C, 92.5% nitrogen, 5.5% carbon dioxide, 2% oxygen. Parasites were cultured in O+ RBCs and maintained in the incubator with daily media change until a parasitaemia of more than 5% ring stages were obtained. The culture was then treated with 5% sorbitol to obtain synchronized ring stage parasites. Forty eight hours after synchronization, the parasites were sub-cultured and parasitaemia was reduced to 1% prior to use in assays.

### Plating of extracts and assay

The stock herbal products were diluted 10 fold to obtain a working solution of 100 μg/ml. This working solution was further serially diluted 4-fold to obtain the concentrations 100, 25, 6.25, 1.56 and 0.39 μg/ml. One hundred microliters of each of the five dilutions were plated in triplicate wells of a 96 well plate. Primaquine at 10 μg/ml and artesunate at 200 ng/ml were used as control drugs in the assays. A 100 μl parasite culture at 1% parasitaemia was added to each treated well. The plates were placed in a Modular^®^ incubating chamber and gassed for 6 minutes. The incubating chamber was then placed in an incubator for 72 hours, after which a thin smear was prepared from each well. The smears were fixed in absolute methanol and stained with 10% Giemsa for 15 minutes. The slides were dried and observed under a compound light microscope using a 100x oil immersion objective lens. The parasitaemia was determined and used to calculate percentage growth inhibition and IC_50_s.

### Culturing of gametocytes

Gametocytes of *P*. *falciparum* were generated as previously reported with minor modifications [18]. A 12.5 ml culture was seeded with a 0.2% parasitaemia, at 6% haematocrit in a T-75 cm^3^ flask (750 μl of RBCs) on day 1, and maintained in the incubator with daily media change and thin smear preparation to monitor parasite growth. From day 4 onwards, media was replaced daily with 25 ml of CPM to reduce the haematocrit to 3%. On days 9–11, 50 mM of N-acetyl glucosamine (NAG) was added to the cultures. On day 12, synchronous gametocytes were purified from the culture using 60% Percoll density gradient centrifugation [19]. The purified gametocytes were put back into culture and after 3–4 hours, half of them were transferred into 96-well tissue culture plates for early stage gametocyte (I-II) assay. The other half of the gametocyte culture was maintained with daily change of NAG-supplemented CPM until day 14, when late stage gametocytes (III-V) were harvested and transferred unto 96-well plates for the late stage gametocyte assay.

### Plating of drugs for gametocyte assay

Herbal products at 100 μg/ml and 1 μg/ml, artesunate at 200 ng/ml and, primaquine 200 ng/ml) and DMSO (1%) were added to triplicate wells of a 96 well plate. Gametocytaemia of the parasite culture was adjusted to 5% by adding RBCs to the culture. Ninety microlitres of either early of late stage gametocyte culture was pipetted into each treated well. The plates were put into an incubating chamber and gassed for 6 minutes. The incubating chamber was then placed in the incubator for 72 hours. Thin smears were prepared from each well and stained with 10% Giemsa for 15 minutes. The slides were dried and observed under a 100x oil immersion objective lens to estimate gametocytaemia.

### Kinetics of herbal antimalarial drugs

Complete parasite medium was prepared in T-25 cm^3^ flasks and supplemented with IC_10_ concentrations of each of the ten herbal products and artesunate (AS). Twelve separate cultures (ten with herbal products, one with AS and one with CPM as control) were set up at 1% parasitaemia and placed in the incubator. Thin smears were prepared from each flask daily, after which media were changed using the same starting media. The parasitaemia and parasite stages were examined under 100x magnification and recorded.

### Statistical analysis

Drug concentration that inhibits asexual *P*. *falciparum* parasites by 50% (IC_50_) were estimated from dose-response curves by non-linear regression analysis using GraphPad Prism^®^ 5.0 software package (GraphPad Software, San Diego, CA, USA). Differences in parasites response to anti-malarial herbal products were estimated by one-way ANOVA and the p value for statistical significance was set at 0.05.

### Ethical clearance

The study was reviewed and approved by the Scientific and Technical Committee and the Institutional Review Board (IRB), both of the Noguchi Memorial Institute for Medical Research (NMIMR), University of Ghana, Legon. Written informed consent was sought from participants of the consumer survey study.

## Results

### Herbal products for treating malaria

One hundred and two licensed chemical shops were surveyed from September to December 2013. The top ten most patronized herbal anti-malarial products [designated as YF, CM, MS, AN, HB, TB, AD, TF, KG and RT] were selected for used in this study (Table 1). Seven of the herbal products were available in 80% of the shops surveyed. The herbal product RT had the highest estimated total sales with an average of 73 bottles sold per shop in a week. Table 1 shows the recommended dosage, active ingredients as well as the alternative uses of the herbal products other than for treating malaria. The most common plant products in the ten herbal products sampled were *Cryptolepis sanguinolenta* and *Azadirachta indica*. Five of the products (YF, CM, MS, HB and KG) contained *C*. *sanguinolenta*, four (YF, TB, HB and TF) contained *A*. *indica* and two products contained both of the two plants (YF and HB). Only three of the products (AN, AD and RT) did not contain these two plants, and three (HB, KG and TF) were exclusive for treating malaria.

### *In vitro* drug sensitivity assessment and gametocyte growth kinetics

**Fig 1:**
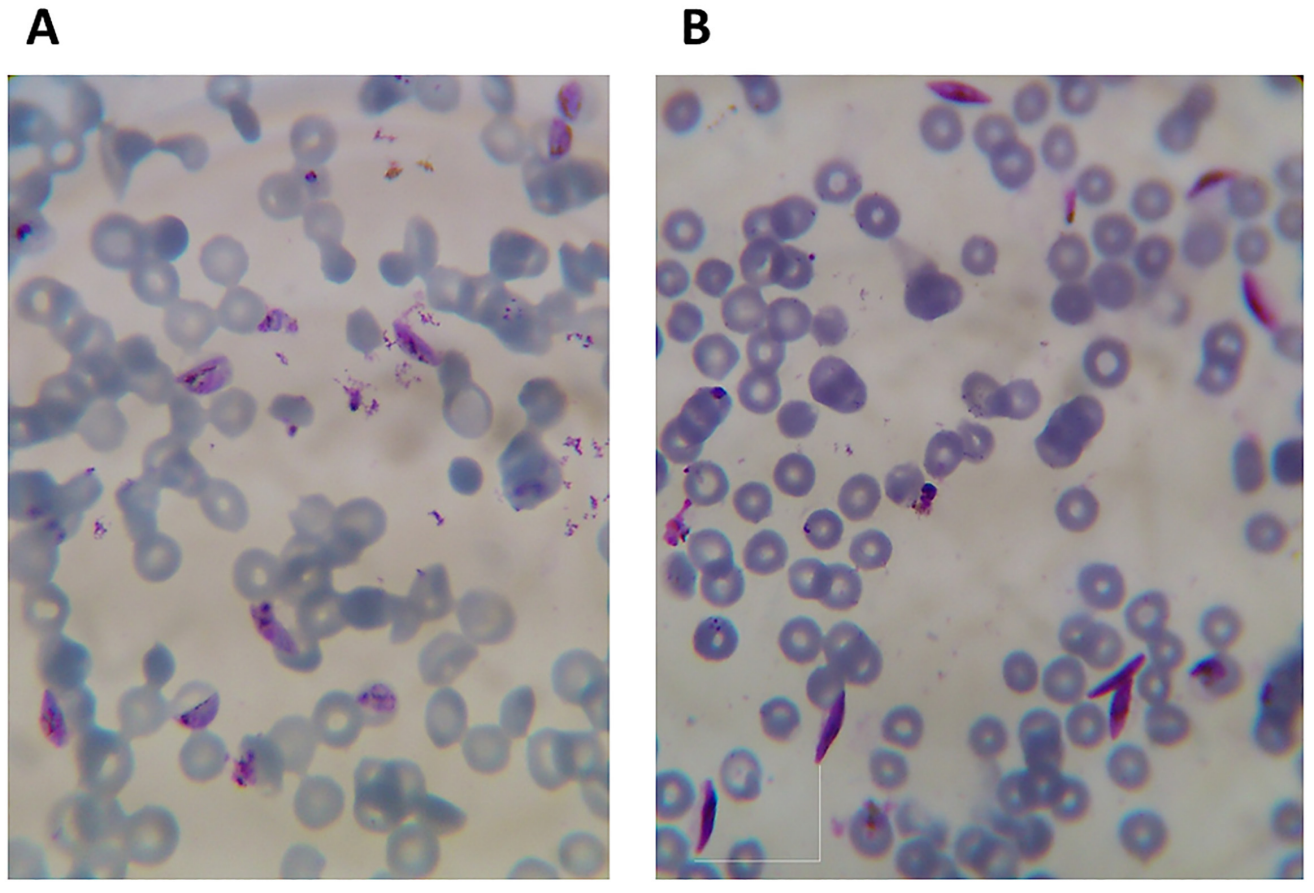
Early (A) and late (B) developmental stages of *Plasmodium falciparum* gametocytes. Gametocytes were harvested after 12 and 14 days of continuous *in vitro* culturing

**Fig 2:**
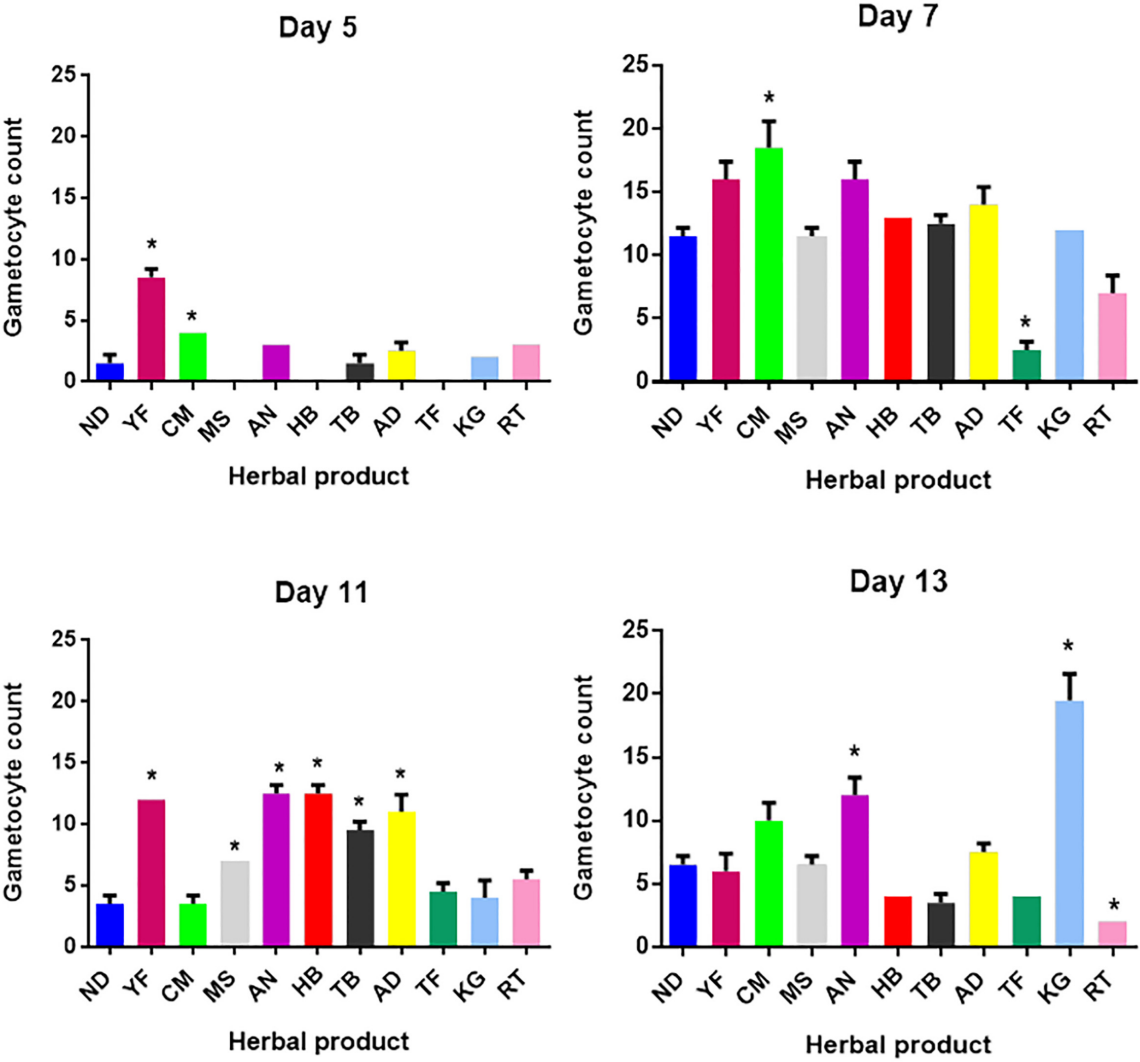
Gametocyte development in the presence of suboptimal herbal product concentration. Asexual *P*. *falciparum* parasites (3D7 strain) were cultured in the presence of suboptimal (IC_10_) levels of each of the 10 herbal products. Cultures were checked for gametocytes on days 5, 7, 11 and 13. ND is the control assay with just culture medium and no herbal product. Asterisks (*) indicate gametocyte counts that were either statistically significantly higher or lower than that of the control (ND). Assays were done in triplicate and error bars represent the standard deviations from at least two repeat experiments.

Of the 10 herbal products tested, five (YF, CM, MS, AN and TF) showed the highest asexual anti-malarial activity with IC_50_ values less than 5 μg/ml (Table 2). Three of these products contain *C*. *sanguinolenta*, two contained *A*. *indica* while one product (AN) did not contain any of these two plants; Gametocytes were generated *in vitro* from asexual 3D7 parasites. Early stage (stage I-II) gametocytes (Fig 1A) and late stage (stage III-IV) gametocytes (Fig 1B) were produced in continuous *in vitro* cultures of asexual parasites after five days and 11 days respectively. After 5 days of exposing the parasites to low doses (below IC_10_ values) of herbal products (Art 0.24 ng/ml, KG 80.87 ng/ml, RT 6.74 ng/ml, TF 2.59 x10^-5^ ng/ml, CM 1.24 ng/ml, MS 1.55 ng/ml, AN 0.86 ng/ml, YF 1.62 ng/ml, HB 83.63 ng/ml, TB 5.56 ng/ml, AD 56.07 ng/ml), cultures treated with YF generated over 5-fold increase in early stage gametocytes as compared with control cultures (ND = no drug, p < 0.05, Tukey post-hoc test). Cultures treated with CM also produced significantly higher numbers of gametocytes as compared to the control culture (*p* < 0.05, Tukey post-hoc test, Fig 2). Although there were no microscopically detectable gametocytes in cultures treated with MS, HB and TF on day 5, HB and MS gametocyte counts were similar to the control cultures by day 7. There was a general increase in the generation of early stage gametocytes by day 7 in all the treated cultures, with CM producing almost 1.5 fold more gametocytes than the control cultures (*p* < 0.05, Tukey post-hoc test, Fig 2). Cultures treated with TF and RT however produced much fewer gametocytes than the control untreated cultures (*p* < 0.05 in each case, Tukey post-hoc test, Fig 2).

**Table 2:** IC_50_values for the different herbal products against asexual parasites.

Herbal product	IC_50_ (ng/ml)
YF	1.41 ± 1.57
CM	1.41 ± 1.49
MS	1.44 ± 1.53
AN	3.37 ± 1.37
HB	12.18 ± 1.30
TB	81.59 ± 1.48
AD	82.25 ± 1.91
TF	3.03 ± 1.45
KG	21.93 ± 1.45
RT	24.58 ± 1.49

Values reported as Mean ± SEM for at least two repeat experiments.

On day 11 we found late stage gametocytes (stage III-IV) in all the cultures; however those treated with YF, AN, HB TB and AD had all produced more than twice the number of gametocytes that we found in the control cultures (*p* < 0.05 in each case, Tukey post-hoc test, Fig 2). Late stage gametocytes produced in cultures treated with CM, TF, KG and RT were not significantly different from the control cultures. By day 13, cultures treated with KG had the highest number of late stage gametocytes, three times higher than gametocytes produced in the untreated control (*p* < 0.05,Tukey post-hoc test, Fig 2), an average of 20 compared to an average of 6 in the controls. Treatment of cultures with herbal product AN produced significantly higher gametocytes than the untreated cultures respectively on day 13 (*p* < 0.05, Tukey post-hoc test, Fig 2). Herbal product RT produced the lowest number of late stage gametocytes, 2 gametocytes compared to 6 produced in the controls by day 13 which was significantly lower than the number of gametocytes found in the untreated control cultures (*p* < 0.05, Tukey multiple comparison, Fig 2).

### Gametocyte growth inhibition assessment

**Fig 3:**
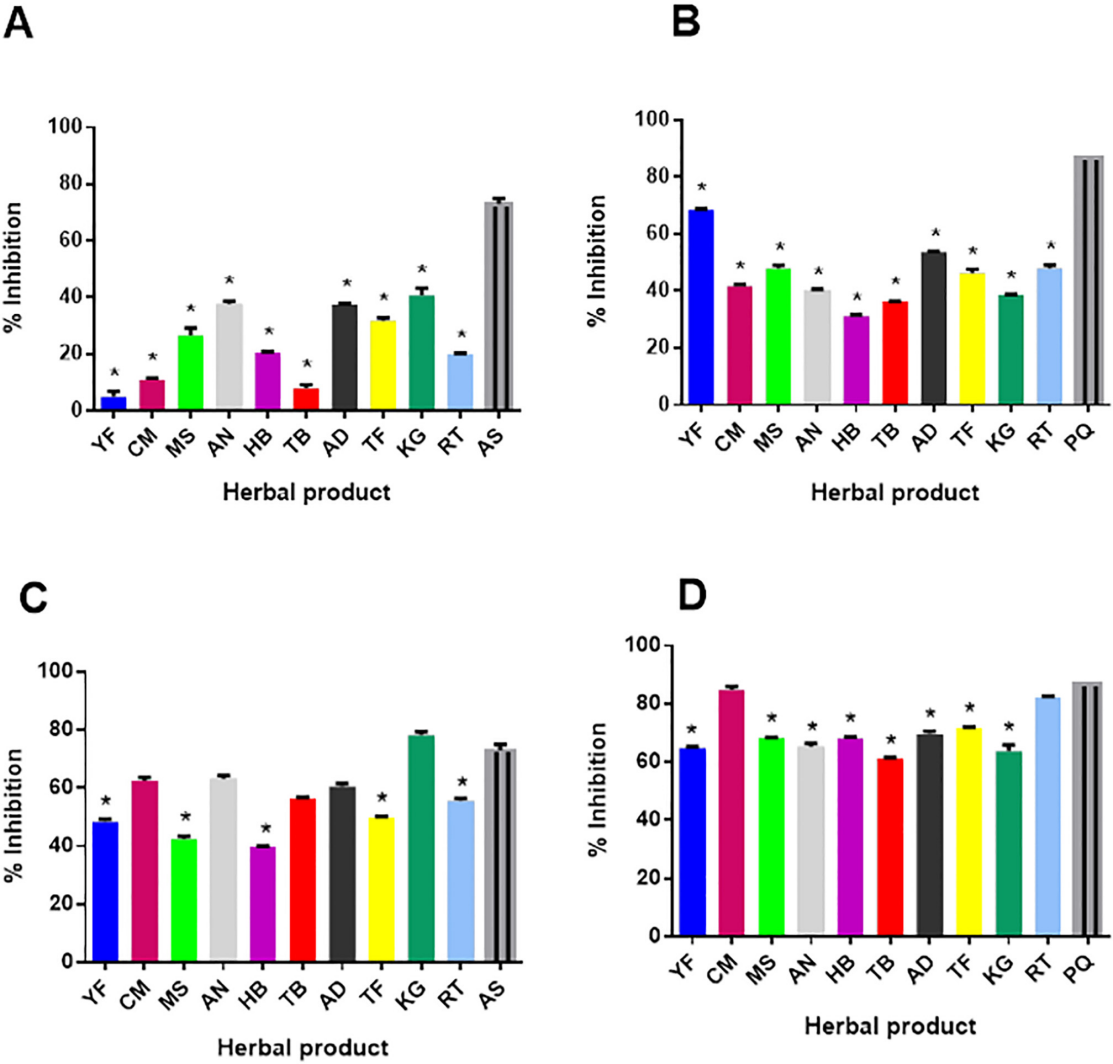
Gametocyte growth inhibition by the 10 herbal products. Asexual *P*. *falciparum* parasites (3D7 strain) were maintained in continuous culture to generate gametocytes. Early stage (day 12) and late stage (day 14) gametocytes were purified and treated with the 10 herbal products for 72 hours. A) early stage gametocytes treated with 1 μg/ml herbal extract, B) late stage gametocytes treated with 1 μg/ml herbal extract, C) early stage gametocytes treated with 100 μg/ml herbal extract, D) late stage gametocytes treated with 100 μg/ml herbal extract. Artesunate (AS) and primaquine (PQ) were added as standard control drugs for the early and late stage gametocytes respectively. For each herbal product, the number of gametocytes remaining after 72 hours was determined by Giemsa stained thin smears and expressed as a percentage of the number in an untreated control setup. Assays were done in triplicate and error bars represent the standard deviations from at least two repeat experiments. Asterisks (*) indicate inhibitions that were statistically significantly lower than that of the respective standard drugs.

At a test concentration of 1 μg/ml, all the herbal products exhibited generally low gametocyte inhibitions that were significantly lower than inhibition with the standard drug Artesunate (AS) at a concentration of 200 ng/ml (p < 0.05 in all cases, Bonferroni multiple comparison test, Fig 3A). Herbal product inhibitions ranged from 5% for YF to 41% for KG and of these herbal products AN, AD, TF and KG exhibited the highest inhibition (> 30%) of early stage gametocytes Late stage gametocytes surprisingly were more inhibited at 1 μg/ml of herbal product than the early stage gametocytes, although inhibitions of all herbal products were still significantly lower than the standard drug Primaquine (PQ) at a concentration of 200 ng/ml (P > 0.05 in all cases, Bonferroni multiple comparison test, Fig 3B). Herbal product HB resulted in the least inhibition of 31% while YF exhibited 68.5% inhibition of the late stage gametocytes. At 100 μg/ml, five herbal products (CM, AN, TB, AD and KG) exhibited inhibitory activities that were statistically similar to that exhibited by Artesunate against early stage gametocytes (P > 0.05, Fig 3C). The least inhibition of early stage gametocytes exhibited by the herbal products was 40% by HB and the highest of 78% was by KG. At least seven herbal products (CM, AN, TB, AD, TF, KG and RT) exhibited greater than 50% early stage gametocyte inhibition (Fig 3C). in contrast, only two herbal products (CM and RT) at 100 μg/ml exhibited inhibitory activities that were statistically similar to that of the standard drug primaquine against late stage gametocytes (P > 0.05, Fig 3D). These two products exhibited greater than 80% inhibition of late stage gametocytes. Herbal product TB exhibited the lowest inhibition (61.5%) of late stage gametocytes at this concentration (Fig 3D). Overall, YF which contains *C*. *sanguinolenta* and *A*. *indica* exhibited the highest gametocydal activity of the tested herbal products since at 1 μg/ml it effectively killed almost 70% of late stage gametocytes; however CM which contains *C*. *sanguinolenta* and *Carapa procera* exhibited the overall best antimalarial activity in that it had the combination of a low asexual stage IC_50_ as well as the highest inhibitory activity against late stage gametocytes in addition to very high inhibitory activity against early stage gametocytes at 100 μg/ml.

## Discussion

The widespread use of artemisinin and its derivatives from the herbal plant *Artemisia annua* in the last decade has increased interest in the potential of locally grown plants to provide new and more potent drugs in the treatment and eradication of malaria. Although artemisinin and its analogues have proven to be a wonder drug for the treatment of chloroquine-resistant malaria, they are effective against the asexual parasite and only the young immature gametocyte [20,21]. Thus there is still a high proportion of malaria patients harbouring highly infectious mature gametocytes after artemisinin treatment in the form of artemisinin-based combination therapy (ACT) [22–24]. There is therefore an urgent need to identify anti-malarial drugs with gametocydal activity to aid in the malaria elimination agenda. With reports of increased and widespread distribution of sub-standard and counterfeit orthodox anti-malarial drugs on the market and the general high patronage of herbal products in Ghana, we set out to identify local herbal anti-malarial products that possess dual activity against the asexual and sexual stages of the malaria parasite. Such herbal products will have both curative and transmission reducing properties that could help in the malaria elimination agenda.

From the survey we conducted in selected chemical shops in the capital of Ghana, we found that the herbal product AN was the most patronized and CM was the least patronized and least available herbal product amongst the 10 selected products. Seven of the herbal products were in addition also very popular for the treatment of either typhoid or jaundice or both. The ability of these products to cure other illnesses is probably due to the fact that a single herbal plant contains several phytochemicals and most of these herbal products tested contain multiple plant components. This thus increases the likelihood of the herbal product curing multiple diseases.

The herbal product CM, which was the least patronized, was however identified as the best anti-malarial product due to its very low asexual stage IC_50_ in addition to its relatively high gametocydal activity (highest activity against late stage gametocytes and the third highest activity against early stage gametocytes at 100 μg/ml). This suggests that patronage of these products may not be directly linked with their antimalarial potency since even products with IC_50_ > 80 μg/ml (TB and AD), and hence expected to have lower efficacy than CM, were much more patronized than CM. Although these herbal products have all been licensed for sale in Ghana, there is very little scientific efficacy data available and this may contribute to the skewed patronage of potentially less efficacious products. Although there are new and improved herbal antimalarial products on the Ghanaian market, people are reluctant to change a particular product as long as they get relief from using that brand [25]. People depend on indigenous herbal products for various reasons including the unavailability of modern healthcare facilities and poverty amongst other factors [26].

Of the five herbal products that contain extracts from *C*. *sanguinolenta*, IC_50_ for activity against asexual parasites increased in the order CM < YF, <, MS < HB < KG. Although we have no idea of the exact quantity of *C*. *sanguinolenta* in each of the herbal products, *C*. *sanguinolenta* has been previously reported to exhibit high antimalarial properties [27, 28]. Coincidentally MS, which exhibited one of the highest anti-malarial activities among the five, contains only *C*. *sanguinolenta*, and has been suggested to be effective against *P*. *falciparum* mono-infections. In addition to *C*. *sanguinolenta*, CM has *C*. *procera*, which is not known to have anti-malarial properties although there has been reports of its anti-filarial properties [29]. YF contains *A*. *indica* in addition to *C*. *sanguinolenta*, and its relatively high antimalarial activity could be a result of the synergistic inhibitory activity that results from the combination of *C*. *sanguinolenta* and *A*. *indica* within the concoction. Anti-malarial synergy has been demonstrated both *in vitro* and in clinical trials of atovaquone and proguanil, where the activity of the combination is up to eight times greater than that of the individual compounds [30, 31]. The other two *C*. *sanguinolenta* containing herbal products, HB and KG had much lower activity against the asexual parasite, and this could be attributed to potential antagonistic effects of components of the additional plants, or that they contained relatively lower concentrations of *C*. *sanguinolenta*. Herbal products HB (containing the plants *C*. *sanguinolenta*, *A*. *indica*, *Alstonia boonei*, *Monodora myristica* and *Xylopia aethiopica*) had much higher activity against the asexual parasite than KG (*Nauclea latifolia*, *P*. *fraternus* and *C*. *sanguinolenta*) and this could be attributed to the added benefit of *C*. *sanguinolenta*. Although herbal product HB contains all the components of TF in addition to three other plants, it exhibited a higher IC_50_ against the asexual parasite compared to TF. This suggests that the other three components might have diluted out the activity of *A*. *indica* and *A*. *boonei*.

Since majority of the local herbal plant extracts are used to treat a variety of ailments, it is possible that people who resort to herbal medicine could continuously have some low levels of the active ingredients of antimalarial products circulating in their blood. This informed our decision to determine the effects of sub-optimum concentrations on asymptomatic malaria patients. Asexual *P*. *falciparum* culturing in the presence of low doses of the 10 herbal products revealed that some herbal extracts increased the commitment signal for gametocyte production. The number of gametocytes produced in the treated cultures was much more than those produced in untreated cultures. Gametocytes were produced from the n-1 cycle in the form of sexually committed merozoites within mature schizonts and we expected to see higher gametocyte counts on day 5 from treated cultures relative to the untreated control cultures, if the herbal products were potent enough to exert pressure on the asexual parasite stages. Although low doses of HB and CM both of which contain *C*. *sanguinolenta* did not generate gametocytes by day 5, low dose of YF, which contains *A*. *indica* in addition to *C*. *sanguinolenta* produced the highest number of gametocytes on day 5. Herbal product KG, which also contains *C*. *sanguinolenta* produced the highest numbers of late stage gametocytes by day 13. This suggests that low doses of *C*. *sanguinolenta* in combination with low doses of *Nauclea latifolia*, *P*. *fraternus* either increases the survival of early stage gametocytes into late stage or increases the commitment of sexual stage parasites from the asexual stage parasites. On the other hand low dose *C*. *sanguinolenta* in combination with low dose *A*. *indica* found in herbal product CM generated high numbers of early stage gametocytes, which were unable to mature into late stage gametocytes. Cultures treated with a low dose of herbal product RT which contains *Aloe schweinfurthii*, *Khaya senegalensis*, *Piliostigma thonningii* and *Cassia siamea* consistently produced low numbers of gametocytes. As of day 7, low dose RT-treated cultures contained one of the least numbers of gametocytes and by day 13, RT-treated cultures produced the lowest numbers of gametcoytes. This suggests that one or a combination of the four plant components could possess high inhibitory activity against gametocytes. Not surprising, none of the other nine tested herbal products contain any plant component common to RT and as such late stage gametocyte production in low doses of all other treated cultures were much higher than in RT-treated culture.

Interestingly two of the products (CM and YF) with the lowest IC_50_ against the asexual parasite were also the ones with the highest early stage gametocyte counts. This could be attributed to an exertion of pressure by these products on the asexual parasites and a subsequent enhancement of sexual stage commitment, validating their efficacy against the asexual stages of the parasite. Low dose of herbal product TF which contains *A*. *indica* and *A*. *boonei* and also exhibited high inhibition against asexual parasites also did not generate gametocytes on day 5, suggesting the combination of the two plants delay the onset of gametocytogenesis and also generate very low gametocyte numbers of both early and late stage gametocytes over the 13 days of the assay. This was however not true for the herbal product MS, which had an IC_50_ comparable to those of CM and YF and the reason for this difference is unclear.

It was interesting but not surprising to realise that not all the early stage gametocytes made it into late stage gametocytes, suggesting the natural death of these parasites during continuous culture. This effect was also observed during the gametocyte inhibition assay as almost 50% of the gametocytes used died after the 72 hours incubation period. For cultures treated with some of the herbal products gametocytes remained detectable for up to two weeks. The data collectively suggests that continuous exposure of asexual stage *Plasmodium* parasites to low concentrations of some of these herbal products, even when used to treat other ailments aside malaria, could enhance malaria transmission by increasing the rate of asexual commitment to sexual stage gametocytes. The minimum complete dose schedule for most of the herbal products is between one to two weeks and often requires the purchase of more than one bottle. For example, the herbal product TB requires the continuous consumption of 7 bottles to cure an episode of malaria. These factors in our opinion are strong contributors to the spread of antimalarial drug resistance as it is highly likely that most people would purchase a single bottle of the herbal product and would not continue the treatment for the duration of the course. This therefore has implications for the preparation and use of these herbal products and makes the determination of the recommended dosage and duration of treatment course very important.

This study confirmed the gametocydal activities of primaquine and artesunate as has been previously described [6, 32]. Eighty percent of the early stage gametocytes that were purified after 12 days of culturing asexual *P*. *falciparum* parasites were confirmed to be stage I and stage II gametocytes by microscopy and about 90% of the gametocytes purified after 14 days of culturing asexual parasites were confirmed by microscopy to be late stages; stage III, stage IV and Stage V gametocytes by microscopy. Gametocytes exposed to 1 μg/ml of the herbal product exhibited inhibitions ranging from 5% by YF to 41% by KG compared to the ND control. Late stage gametocyte inhibition ranged from 31% by HB to about 68.5% by YF at 1 μg/ml. It was interesting to note that at 1μg/ml, YF exhibited some inhibition of late stage gametocytes but had relatively no effect on the early stage gametocytes. This is not surprising as some drugs have been found to affect young asexual ring stage parasites but not the late asexual parasite [33]. With this in mind it is possible that some herbal products will have high inhibitory activity against either late or early stage gametocytes and not the asexual stages, or vice versa.

Inhibition of gametocyte survival was more significant at 100 μg/ml, with early stage gametocyte inhibition ranging from 40% in HB to 78% in KG and late stage gametocytes ranging from 63.5% in KG to 85% in CM. Two herbal products tested, CM-85% and RT-82% exhibited greater than 80% efficacy at clearing late stage gametocytes at 100 μg/ml. These products surprisingly do not contain any common plant extract, suggesting the combination of extracts in RT yields similar late stage gametocyte inhibition as those found in CM. Herbal product KG at 100 μg/ml exhibited almost 80% inhibition of early stage gametocytes, despite it having one of the poorest asexual parasite inhibitions. Interestingly all the herbal products we tested exhibited at least 60% growth inhibition of late stage gametocytes at 100 μg/ml. However, at 1 μg/ml, only herbal products YF and AD exhibited > 50% inhibition of late stage gametocytes. None of the other herbal products had greater than 40% inhibition of either late or early stage gametocytes at 1 μg/ml. Our findings show that the herbal products exhibited very low inhibition of gametocytes at 1 μg/ml, the highest inhibition of 39% was exhibited by KG. KG was the only product that displayed higher activity against early stage gametocytes (78%) at 100 μg/ml than late stage gametocytes (63%) at the same concentration. This was also seen at the 1 μg/ml concentration, where inhibition of late stage gametocytes by KG was lower (38.5%) than inhibition of early stage gametocytes (41%). This finding suggests that the biology of early and late stage gametocytes are different such that some drugs as well as these herbal products could affect one and not the other. At 10 μg/ml, PQ exhibited 86.5% inhibition of late stage gametocytes whilst AS at 200 ng/ml inhibited late stage gametocytes by 50.5%.

We plated the assay at an initial gametocytaemia of 5%, however after the 72 hour incubation, the gametocytaemia reduced to 2.7% in the ND control wells for the early stage gametocytes and 2.2% in the late stage gametocytes, suggesting about 50% loss (45% and 55% for early and late stage gametocytes, respectively). Though this is high we expect it to be uniform across the plates and as such not affect the results of the assay. We are uncertain of the proportions and amounts of each active plant extract that are contained in these herbal products and relied only on the product label and packaging for information.

## Conclusions

The ideal antimalarial product in the era of malaria elimination and eradication must target both the asexual and the sexual stage parasites. Herbal products CM and TF exhibited good antimalarial properties as they targeted both the asexual parasites as well as mature gametocytes. A product such as RT which had very high activity against the mature gametocytes had very little activity against the asexual parasite. We believe that adding either *C*. *sanguinolenta* or *A*. *indica* which have proven to exhibit high activity against the asexual parasite to the composition of RT could enhance the overall antimalarial properties of RT and transform it into an ideal antimalarial herbal product against both sexual and asexual stage parasites.
